# The structure of complexes between zinc(ii) cations and histidine-rich repeats from the unstructured N-terminal domain of human prion protein

**DOI:** 10.1039/d5ra04584c

**Published:** 2025-10-15

**Authors:** Michał Nowakowski, Joanna Wolak, Maciej Gielnik, Adam Piotrowski, Igor Zhukov, Justyna Żygowska, Aneta Szymańska, Marta D. Wiśniewska, Wojciech Bal, Sebastian K. T. S. Wärmländer, Maciej Kozak, Wojciech M. Kwiatek

**Affiliations:** a Department of Chemistry, Center for Sustainable Systems Design (CSSD), Faculty of Science, Paderborn University Warburger Straße 100 33098 Paderborn Germany michal.nowakowski@upb.de; b Faculty of Physics, Adam Mickiewicz University Uniwersytetu Poznańskiego 2 Poznań 61-614 Poland; c Department of Molecular Biology and Genetics, Aarhus University Nordre Ringgade 1 8000 Aarhus Dennmark; d Institute of Biochemistry and Biophysics Polish Academy of Sciences Pawińskiego 5a 02-106 Warsaw Poland; e Department of Chemistry, University of Gdańsk Jana Bażyńskiego 8 80-309 Gdańsk Poland; f Chemistry Section, Arrhenius Laboratories, Stockholm University 106 91 Stockholm Sweden; g CellPept Sweden AB Kvarngatan 10B 118 47 Stockholm Sweden; h Smaug Beamline, SOLAIS National Synchrotron Radiation Centre Czerwone Maki 98 30-392 Cracow Poland; i Institute of Nuclear Physics Polish Academy of Science Radzikowskiego 152 31-342 Cracow Poland

## Abstract

Prion protein (PrP^C^), a well-known protein pathogenic agent, consists of an ordered C-terminal domain and an unstructured N-terminal tail. The N-terminal region includes a highly conserved region consisting of four octarepeat sequences PHGGGWGQ (in short, octarepeats). These octarepeats are capable of binding metal ions such as Cu(ii) and Zn(ii). In this study, XAS and FTIR experiments revealed the specific stoichiometry and characteristic features of the Zn(ii)-binding site in octarepeats. In the presence of Zn(ii) ions, the octarepeat peptide can self-assemble and form fibrils. Although fully developed aggregates are visually distinct, their base PrP–Zn(ii) complex geometry remains the same everywhere – Zn(ii) is coordinated by N atoms from His residues in the octahedral structure, with axial water molecules being preferred. The coordination of Zn(ii) ions promotes β-sheet formation in the secondary structure of the octarepeats, reducing the structural disorder level and favoring oligomerization in aqueous solutions—the results clearly evidence that Zn(ii) ions have potential to promote neurodegenerative diseases *via* unwanted interactions with PrP.

## Abstract 2

Prion protein (PrP^C^), a well-known protein pathogenic agent, consists of an ordered C-terminal domain and an unstructured N-terminal tail. EXAFS shows it coordinates Zn^2+^ in octahedral geometry with four His groups involved.

## Graphical abstract

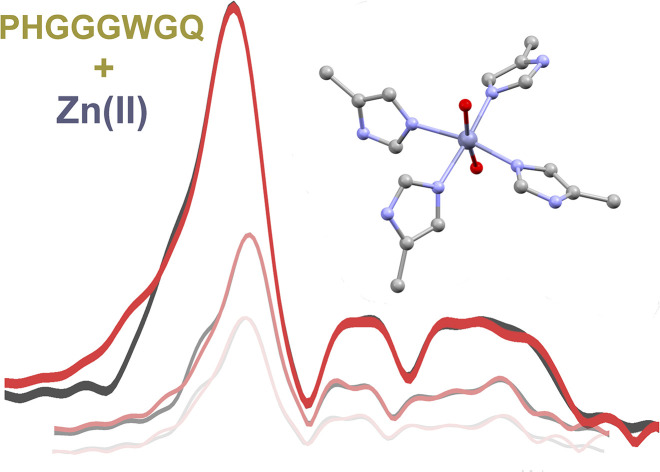

## Introduction

Prion protein (PrP^C^), in its standard, physiologically correct cellular form, is a 23 kDa glycoprotein that is found in the cell membranes of many tissues and is particularly abundant in the nerve cell membrane of the central nervous systems of mammals and many birds.^1,2^ The structure of human prion protein and its mutants and variants from other organisms (elk, horse, mouse, pig, cat, *etc.*) has been partly characterized using NMR spectroscopy and protein crystallography.^3–13^

The PrP molecule consists of two domains: the unstructured and flexible N-terminal domain containing four consecutive octarepeats (octarepeat sequence: PHGGGWGQ)^14,15^ and the C-terminal domain with a predominantly α-helical structure.^3,5–13^ The prion protein is anchored to the outer surface of the cell membrane *via* glycosylphosphatidylinositol located at the C-terminus.^16^ Despite many years of intense structural studies, the physiological function of PrP^C^ in healthy tissues is still ambiguous; however, its ability to bind Cu(ii) and Zn(ii) ions and other divalent cations has been characterized.^17–21^

The relationship between PrP and neurodegenerative diseases such as spongiform encephalopathies^1,2,22^ has also been known for decades. These diseases, which degenerate the human and animal nervous systems, are caused by massive accumulation of incorrectly folded (containing β-sheet-rich secondary structure) prion deposits (PrP^Sc^). Prion diseases are incurable. They have a long incubation period but a fairly rapid disease progression once the first symptoms appear, leading to the patient's death.^22^

It is believed that the tertiary structure of the prion protein, modulated by Zn(ii) ions, may induce the neurotoxicity of PrP^C^.^15,23^ However, it should be noted that the sequences of zinc- or copper-binding amino acid residues are not unique to prion proteins and have also been identified in many other proteins. These motifs are usually associated with the enzymatic activity of these proteins.^24^ Typical Cu(ii) ion binding motifs contain different histidine, sometimes cysteine, and/or methionine repeats.^25,26^ Similar cysteine- and histidine-rich sequences form zinc-binding motifs.^27,28^ While the Cu(ii) ion has the strongest coordination affinity for the N-terminal domain of PrP^C^, Zn(ii) ions can compete with Cu(ii) when conditions are favourable enough.^17,29^

The unstructured N-terminal fragment of PrP assumes a flexible polypeptide chain conformation that allows effective transition and group 10 metal ion binding.^1,30^ In our previous studies, we have shown that Zn(ii) coordination in the peptide (PrP^58–93^) containing the PHGGGWGQ sequence from the N-terminal part of the human PrP mediates formation of β-sheet secondary structure^31^ and promotes formation of amyloid-like fibrils,^32^ suggesting a possible pathway for pathological aggregation, while in the full length PrPC, Zn(ii) can stabilize α-helix 3 from the C-terminal domain.^31^ We previously proposed models for Zn(ii) binding in the octarepeat region; however, the Zn(ii) coordination mode is of significant interest due to potential role in protein misfolding, aggregation and development of prion diseases. Therefore, the main purpose of this work was to study the structure and stoichiometry of Zn(ii) complexes with peptides forming fibrils containing the octarepeats using X-ray absorption spectroscopy (XAS) and Fourier transform infrared spectroscopy (FTIR).

## Experimental

### Sample preparation

For XAS measurements, the inorganic and organic references used were ZnO, ZnSO_4_ × 7H_2_O, Zn(ii) ions coordinated by imidazole, and Zn(ii) ions coordinated by HSA (human serum albumin, HSA–Zn). All inorganic ingredients were purchased from Sigma-Aldrich. The Zn(ii)–imidazole complex was prepared by mixing concentrated ZnCl_2_ and imidazole solutions in water. The pH was adjusted to 7.4 by adding concentrated NaOH. The final concentrations were 50 mM imidazole and 12.5 mM Zn(ii). The solution was lyophilized, and the obtained powder was used for measurements. A synthetic peptide designed based on the N-terminal sequence of human prion protein (*hu*PrP) constituted the third type of sample. The PrP^58–91^ peptide sample used for XAS experiments was synthesized and purified by CASLO ApS, c/o Technical University of Denmark (Kongens Lyngby, Denmark). The peptide containing residues 58–93 from the *hu*PrP sequence, denoted PrP^58–93^, was synthesized as previously described and used in this work for FTIR experiments.^31,32^ The Zn(ii) complexation with PrP^58–93^ or PrP^58–91^ was obtained by careful titration of the peptide with ZnCl_2_. The PrP–Zn(ii) samples contained different Zn(ii) concentrations with respect to the initial protein concentration. For the initial PrP^58–91^ concentrations of 400 μM and 1200 μM, Zn(ii) ions were titrated at ratios of 1 : 1, 1 : 4, and 1 : 10 to achieve higher concentrations of the holopeptide. The final samples subjected to XAS experiments correspond to octarepeat fibrils formed by incubation of the peptide with Zn(ii) for 24 h at 37 °C (ref. 32) and lyophilized. We did not expect any significant influence of lyophilization on the Zn(ii) local environment. Moreover, the use of lyophilized samples significantly reduced the risk of radiation damage, most of which occurs due to solvent ionization. All measured samples were in the form of pellets placed on Kapton foil.

### Geometry optimizations

Initial structures for geometry calculations were built using Avogadro software. Geometries were optimized using the Orca 5.0.3 package.^33^ For calculations, the composite approach PBEh-3c^34^ was applied with the def2-mSVP basis set and def2/J auxiliary basis sets^35^ along with the geometrical counterpoise correction gCP^36,37^ and the atom-pairwise dispersion correction with the Becke–Johnson damping scheme (D3BJ).^38^ The input file and final geometries are available in the SI. The third initial model was obtained *via* an *in silico* molecular dynamics experiment, which was further optimized as above. In all cases, due to the initial failure, a stepwise approach was taken, with more rigid convergence conditions applied. Numerical frequencies were calculated and checked for the absence of negative values to confirm a minimum-energy structure.

### XAS measurements and analysis

Zn K-edge XAS measurements were carried out at the P64 beamline in DESY (Hamburg). The total photon flux on the sample was approximately 3 × 10^12^ ph per s. Energy selection was performed by a Si(111) double crystal monochromator (DCM) with a step size of 0.3 eV. Data were acquired using a TFY PIPS X-ray detector (Canberra GmbH). During the experiment, the samples were cooled to *T* = 10 K in a liquid He flow cryostat (Oxford). We estimate that the beam energy uncertainty due to the step size should be half of it, *i.e.*, Δ*E* = 0.15 eV in the near-edge region. An extended radiation damage experiment was performed (Fig. S1). Neither significant self-absorption (SA) nor significant radiation damage effects were observed. Initial data reduction, which includes both background subtraction and normalization, was performed with the Athena program, whereas the detailed analysis based on multiple scattering (MS) theory was performed with the use of Artemis software.^39^

During Extended X-ray Absorption Fine Structure (EXAFS) analysis, for each complex, multiple coordination schemes were considered. For example, the SOD crystal structure (see Results section for details) contains two distinct Zn centres, and both were tested during analysis. Moreover, different coordination environments were built and optimized *via* the theoretical approach for the initial fitting attempts. The initial fit of the 1st coordination shell was performed in the range of 1–2.3 Å using single scattering paths Zn–N or Zn–O for some starting models. In further analysis, the 2nd and 3rd coordination shells were fitted straight with single and multiple scattering paths, depending on the path, initial shape, and positions in the path list. A qualitative comparison between Fourier-Transform EXAFS (FT EXAFS) and computed paths was conducted to determine which path could be a potential candidate for fitting. Protein samples are not symmetric, unlike small molecules or crystals, and single scattering paths do not always dominate in the EXAFS. Thus, the single scattering path selection approach was abandoned for higher coordination shells, and essential single and multiple scattering paths were fitted together. The selection of multiple scattering paths was made upon their importance in MS calculations results. Each path was added separately, once no single scattering paths were available, and its influence on the final fit was tested in terms of relative *χ*^2^ change. Additionally, the selection was limited to the triangle and colinear multiple scattering paths as they occur with rigid angles (His rings) and linear geometries, respectively.

EXAFS has limited sensitivity to coordination numbers of light elements and provides average coordination numbers in the system. Given that, coordination numbers were refined once robust fitting model was already established using default values from the structural model. The refinement was sone with all other parameters (*i.e. E*_0_, *σ*^2^, Δ*R*, and *C*_3_) fixed due to the limitation on total parameter count.

### FTIR measurements and analysis

For FTIR studies, a solution of PrP^58–93^ peptide at a concentration of *c* = 595 μM in 10 mM HEPES buffer, pH 7.3, was prepared. The measurements were performed using a Bruker Tensor 27 spectrometer equipped with an MCT detector using the platinum ATR attachment. A drop of peptide solution (20 μL) was applied to the diamond crystal and was allowed to dry completely. Then, FTIR spectra were recorded in the range of 4000–400 cm^−1^ with a spectral resolution of 2 cm^−1^. Each measurement was performed with 512 scans. For the study of zinc chloride PrP^58–93^ complexes, an appropriate volume of zinc chloride solution was added to the prion peptide solution and mixed gently. The samples were also allowed to dry completely, and then FTIR spectra were collected.

FTIR spectra were subjected to background correction and smoothing with the Savitzky–Golay (10-point mode) procedure. The analysis was focused on the amide I band (1720–1580 cm^−1^). To determine the secondary structure in the tested peptide solutions, this band was deconvoluted by the second derivative method.^40^

## Results and discussion

### Qualitative XAS data analysis

**Fig. 1:**
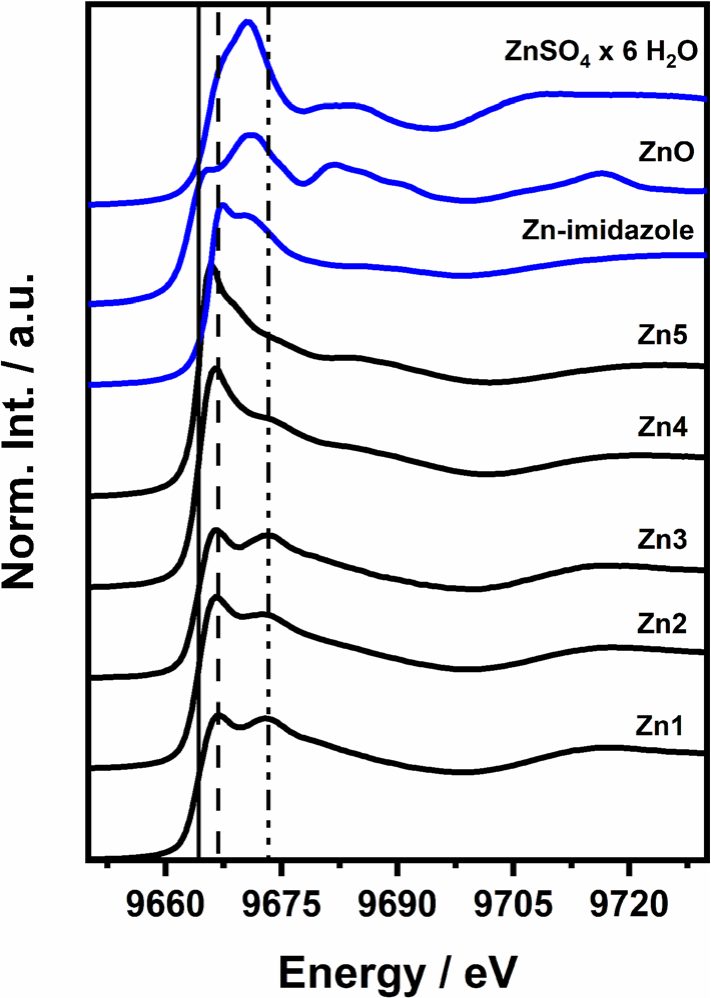
XANES spectra of five Zn(ii) complexes of PrP^58–91^ peptide (black): Zn1 (1 : 1, 400 μM), Zn2 (1 : 4, 400 μM), Zn3 (1 : 1, 1200 μM), Zn4 (1 : 4, 1200 μM), Zn5 (1 : 10, 1200 μM) and references (blue): ZnSO_4_ × 6H_2_O, ZnO and Zn–imidazole complex. Vertical lines indicate characteristic energies: 9664 eV (solid), 9667 eV and 9673 eV.

The data were calibrated in relation to the first inflection point in the Zn foil XAS spectrum (9659 eV). The spectra were significantly different for each concentration of Zn in the sample and the different protein-to-Zn ratios. The results for the protein samples and references are presented in Fig. 1 with samples denoted as Zn1 (1 : 1, 400 μM), Zn2 (1 : 4, 400 μM), Zn3 (1 : 1, 1200 μM), Zn4 (1 : 4, 1200 μM), and Zn5 (1 : 10, 1200 μM). No pre-edge peak was recorded for a single Zn centre-containing spectrum. This observation is consistent with the fact that Zn(ii) has a closed-shell d^10^ electronic configuration that prevents transitions to 3d orbitals. While it is possible that the metal-to-ligand charge transfer could partially empty the 3d shell,^41^ it is not the case here, and the first possible transition, which is allowed, is a dipole 1s → 4p transition at the edge position. The edge position (solid vertical line in Fig. 1) is similar for all PrP^58–91^–Zn(ii) samples. Similarly, the white line position is approximately 9667 eV for the protein spectra (dashed, vertical line in Fig. 1). For spectra Zn1–4, there is an additional 1s → 4p feature at 9673 eV in the edge region (dash-dotted vertical line in Fig. 1). Strong peaks closely related to each other at the energy scale in the white line region of the spectrum might suggest a metal–metal (Me–Me) interaction. In metalloprotein samples, metal ions are suspended in a matrix of light elements, and each heavy atom (Zn, in our case) is expected to be clearly visible. The absence of a strong Zn–Zn interaction peak in the |*χ*(*R*)| signal is evidence that Zn occupies a mononuclear site and is coordinated only by light atoms (N, O, S)^42,43^ (see Fig. S2B in the SI). For all samples, the Fourier transform of the *χ*(*k*) signal revealed a significant influence of imidazole groups in the range of 2.75–4.0 Å (ref. 43) (see Fig. S2C in the SI).

The edge position is a good marker of the absorbing ion's physical oxidation state due to its dependence on the effective charge of its nucleus. Empirically, in 3d metal complexes, each increase in the oxidation state by +1 equals approximately 1–2 eV of the energy shift when the absorbing atom is in a similar ligation environment.^44^ In the spectra of PrP^58–91^–Zn(ii) complexes in Fig. 1, the edge positions were read at the first point equal to 0 in the 2nd derivatives of the spectra. They represent four different types of ligation environments. PrP^58–91^ peptide–Zn(ii) complexes have the same absorption edge position in the range of the measurement uncertainty, and their average *E*_0_ is 9664.36(15) eV. The difference is 0.75(30) eV, which in this case demonstrates how strong an impact a different geometry can have on Zn ions with the same ligand types. The structure of PrP^58–91^ samples could be placed in the middle, between the well-ordered and tight ZnO crystal with a distorted *C*_3v_ structure (locally *T*_d_, with good approximation,^45^ Fig. S3A) and the ZnSO_4_ heptahydrate structure, where Zn(ii) is coordinated in an *O*_h_ structure (Fig. S3B). Interestingly, the referential imidazole–Zn(ii) complex perfectly overlaps with the peptide sample's positions, suggesting substantial similarity between the compounds. Additionally, it is worth mentioning that the four closest S atoms in ZnSO_4_ heptahydrate are positioned in pairs at 4.413 Å and 4.771 Å and are fully coordinated by O atoms; thus, only the coordination geometry and number of O atoms affect the edge position difference between ZnO and ZnSO_4_ × 6H_2_O. In conclusion, the Zn(ii) ion in the PrP^58–91^–Zn(ii) sample is in a distorted tetrahedral, pyramidal, planar, or octahedral geometry with two distant axial O atoms, which is expected to exhibit an edge energy lower than that in the symmetric *O*_h_ structure and higher than that of *C*_3v_ with light elements as its ligands. This may happen, for example, due to a different number of ligands or less electronegative ligands in the 1st coordination shell of Zn(ii). Despite the differences in the absorption edge positions, the Zn(ii) ion possesses a formal +2 oxidation state in all discussed compounds. Since the ligation environment is expected to remain similar for all complexes, the edge position differences reflect variations in the local geometry around Zn(ii).

### The LCF analysis

The Zn(ii) binding in the N-terminal region of PrP leads to a mixture of binding site motifs.^46^ Following the gradual changes in the PrP^58–91^–Zn(ii) spectra in Fig. 1, we conducted a linear combination fitting (LCF) procedure to quantitatively and qualitatively describe the changes observed in the spectra. The HSA–Zn(ii) protein complex represented a common nitrogen/oxygen Zn(ii) binding site in unstructured proteins. This site contains two imidazole nitrogens coordinated to the Zn(ii) ion. It has been described as a functional and structural Zn(ii) ion source in the system.^47,48^ The possible local coordination environments of Zn(ii) in HSA–Zn(ii) complex are shown in Fig. S5. The imidazole–Zn(ii) complex was selected to model a histidine-rich bonding environment, while the ZnCl_2_ compound was chosen as an inorganic reference, as it was used during the sample preparation. The following peptide–Zn(ii) complex samples were subjected to the LCF study: Zn1, Zn2, Zn3, Zn4, Zn5, with total peptide concentrations and PrP : Zn(ii) ratios of 400 μM and 1 : 1, 400 μM and 1 : 4, 1200 μM and 1 : 1, 1200 μM and 1 : 4, 1200 μM and 1 : 10, respectively. The Zn1 and 3 had the lowest Zn(ii) concentrations and identical XAS spectra, but represented different final concentrations of the complex. Therefore, these XAS spectra were selected as references, representing most likely the single type of “specific” Zn(ii) binding site. All LCF procedures were performed for the incident energy range of 9654–9729 eV with fixed *E*_0_ values. All obtained *R*-factors had values lower than 0.05, with all *χ*^2^ values lower than 1. Table 1 summarizes the LCF results. Interestingly, none of the obtained fits contained HSA–Zn(ii) contribution. In the Zn2 (1 : 4 protein to Zn ratio), all Zn ions were bonded, and two Zn binding fractions were present with no inorganic compounds: Zn1 and imidazole–Zn(ii) complex. On the other hand, in Zn4 and Zn5, an inorganic contribution to the XAS spectrum, was present. Additionally, Zn5 has the highest histidine-rich fraction and the smallest specific Zn(ii) binding site component. This suggests the possible multi-Zn coordination scheme caused by the excess of Zn(ii) ions. The observed results could also be related to the relative ratios of Zn(ii) binding modes. The LCF results confirmed that the spectra of Zn1 and 3 consisted of one dominant contribution from the Zn(ii)-specific binding site. The LCF plots are presented in the SI. The detailed structure of the specific binding site was further characterized by EXAFS analysis.

**Table 1:** LCF results. Fitted corresponding data are shown in Fig. S4. Contributions from HSA–Zn(ii) were insignificant and have been omitted for clarity

Reference samples	ZnCl_2_ × 4HO	Imidazole–Zn(ii)	Zn1	Zn3	Zn4	Weight sum
Zn2	—	0.258(17)	0.776 (25)	—	—	1.034(42)
Zn4	0.308 (64)	0.464(27)	—	0.371 (36)	—	1.143 (127)
Zn5	0.285 (72)	0.655(36)	—	0.114 (42)	—	1.054(150)
Zn5	—	0.518 (42)	—	—	0.554(45)	1.072 (87)

### *Ab initio* calculations

**Fig. 2:**
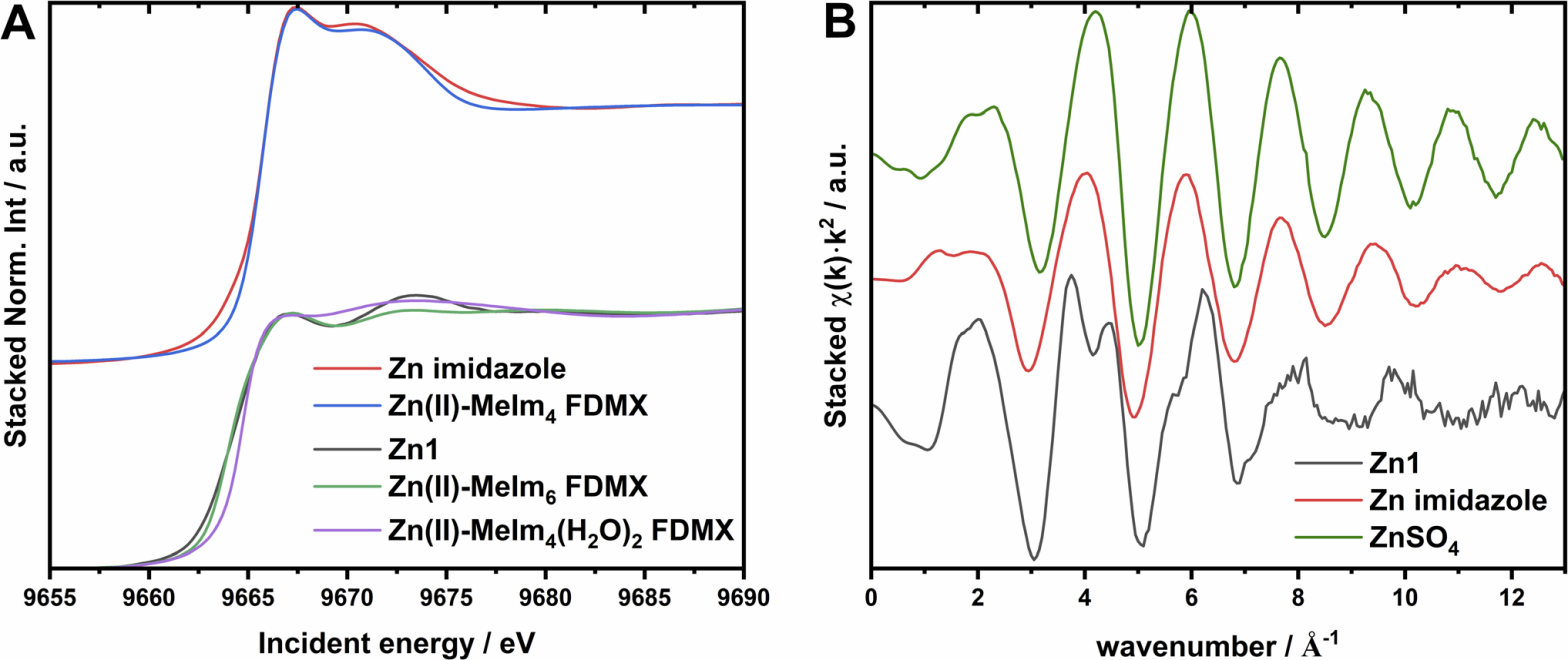
(A) – XANES calculations using FDMX for Zn(ii)–(MeIm)_4_, ZnIm_6_, and Zn(ii)–(MeIm)_6_ modified according to the EXAFS fitting results basing on ZnIm_6_ model, along with corresponding experimental spectra of Zn(ii)–(MeIm)_4_ complex and Zn1; (B) – EXAFS spectra of the Zn1, Zn–imidazole complex, and ZnSO_4_ reference.

The low ligand-field stabilization energy due to a full 3d^10^ shell leads to multiple Zn(ii) structural configurations. The final Zn(ii) environment and complex symmetry are often determined by external factors, for example, thermodynamic, rather than orbital symmetry of the ion.^49^ However, it is well-known that ligand field strength not only affects partially filled valence d orbitals in metals, but also other, higher-lying states. This is particularly evident in the Zn(ii)–imidazole complexes, where the same ligation environment and different symmetry lead to distinct changes in K-edge XAS.^50^ In the case of Zn(ii) ion, the symmetry-related XAS spectral changes are limited to the main edge features and EXAFS region, due to the lack of pre-edge peaks. Fig. 2A shows overlapping Zn K-edge XAS spectra of Zn(ii)–(MeIm)_*x*_(H_2_O)_6−*x*_, Zn1 complexes and calculated Zn(ii)–(MeIm)_4_ and Zn(ii)–(MeIm)_6_ XAS spectra in FDMNES software,^51,52^ based on 3D structures of Zn(ii) coordinated by six 1-methylimidazole molecules published elsewhere.^50^ Literature presents many possible 4-fold and some 5-fold coordination schemes of Zn(ii) in proteins. For example, a square-pyramidal coordination scheme that includes an additional H_2_O molecule perpendicular to the planar coordination system formed by 2O (–COON–) + 2N (his) atoms was reported for Human Serum Albumin coordinating Zn.^53^ 4-Atom coordination is often preferred for the resting state enzymes and metallocomplexes of Zn.^54^ Another example is superoxide dismutase and its structure.^55^ The *hu*PrP^C^ protein with Cu(ii)/Zn(ii) catalytic/structural ions has been reported to act like an SOD-like enzyme, with Zn(ii) increasing the SOD-like catalytic activity.^56,57^ The low energy barrier between hexacoordinated and tetracoordinated Zn motifs implies the need for a careful coordination symmetry evaluation not only for the PrP^58–91^–Zn(ii), but also for the imidazole-based reference complex. The calculations show that most likely, the Zn(ii)–imidazole XAS spectrum represents the tetrahedral geometry pattern of the Zn(ii)–(MeIm)_4_ complex, while the Zn(ii)–(MeIm)_6_ spectrum resembles the PrP^58–91^–Zn(ii) XAS more. The calculation reveals that two edge features at 9667 eV and 9673 eV in the Zn1 spectrum and 9666.4 eV and 9669 eV in Zn(ii)–(MeIm)_6_ are due to the unoccupied 4p_3/2_ and 4p_1/2_ sublevels. Since the Zn1 spectrum represents the unique binding site for the PrP–Zn(ii) complex, and the Zn(ii)–(MeIm)_4_ Finite Difference Method for XAFS (FDMX) spectrum does not resemble it, we have calculated *ab initio* spectra of Zn(ii)–(MeIm)_6_ and Zn(ii)–(MeIm)_4_(H_2_O)_2_. In both cases, there is a significant similarity between the theoretical spectra and the experimental one of Zn1, proving the octahedral coordination geometry of the Zn(ii) ion. However, the model spectra have their limitations, and a more detailed EXAFS analysis is required.

### EXAFS analysis of imidazole complex

We start the EXAFS analysis with a qualitative comparison of the XAS oscillatory parts of the Zn(ii)–MeIm_4_, Zn1, and ZnSO_4_ in H_2_O. Note that in water solution Zn(ii) is coordinated by 6H_2_O molecules. Fig. 2B shows EXAFS signals in the range of 2.5–13.0 Å^−1^. The oscillatory functions of 4N and 6O coordination environments are close in shape, do not exhibit split characteristics for Zn1 EXAFS around 3–6.5 Å^−1^, and, as expected, slightly differ due to the phase shift. Most likely, it is due to the one-atom-type coordination system. A similar conclusion can be drawn by comparing the absorption edge shapes: Zn–imidazole and ZnSO_4_ have monotonic and sharp white line rises, indicating no additional 2p admixtures to the Zn 4p densities.

**Fig. 3:**
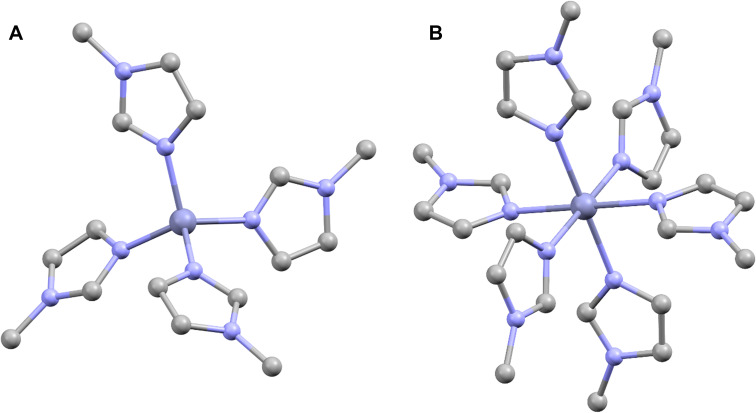
DFT-optimized geometry model structures for EXAFS spectra analysis of Zn(ii)–imidazole complex: (A) tetrahedral Zn(ii)–MeIm_4_; (B) octahedral Zn(ii)–MeIm_6_. Hydrogen atoms were omitted for clarity.

**Fig. 4:**
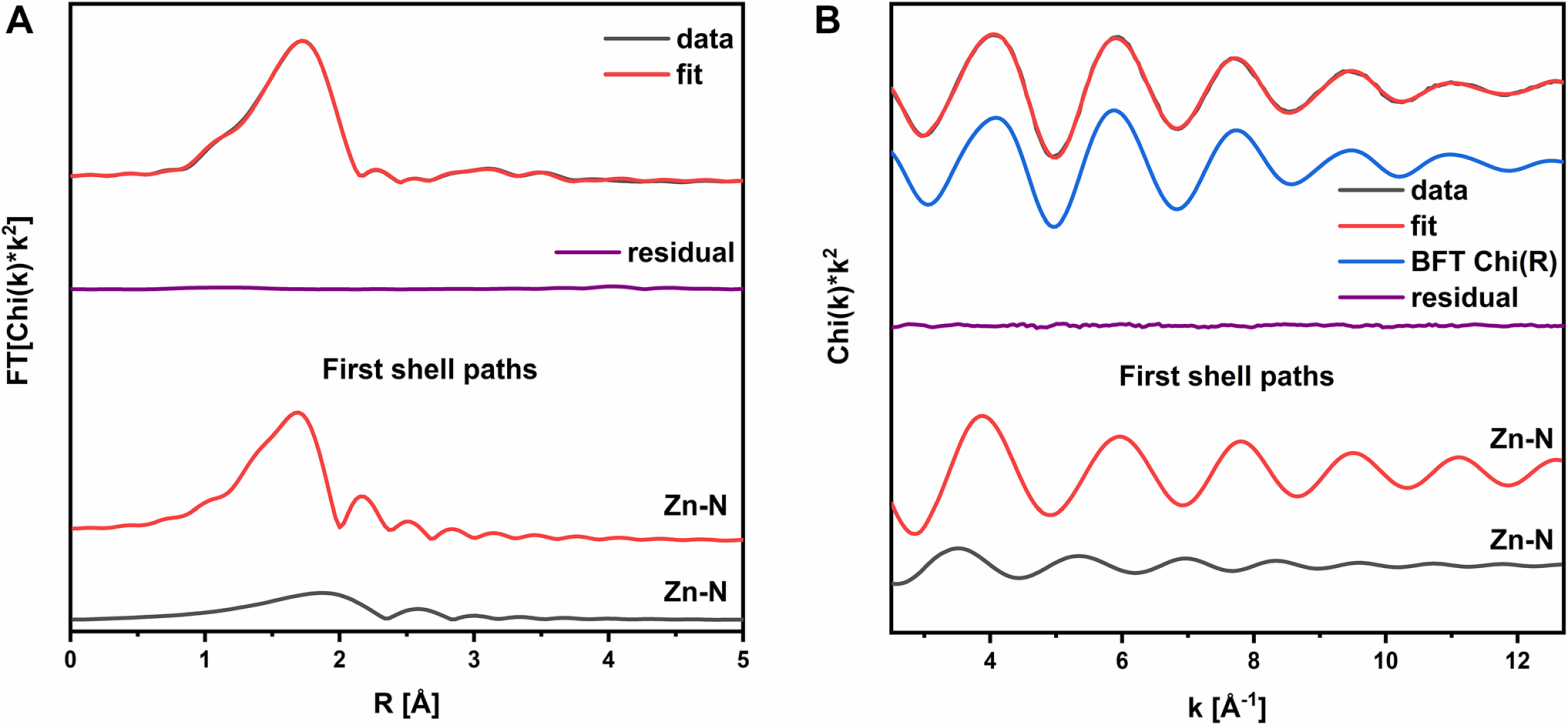
EXAFS fitting results for Zn(ii)–imidazole complex in the form of Zn(ii)–MeIm_4_: (A) *R* space with Chi(*R*) and fitted function compared to the residual function and first shell paths; (B) *k*-space with EXAFS signal (*k*) and fitted function compared to the backward Fourier transform-filtered EXAFS (FFT(Chi(*R*))), residual function and first shell paths.

In the first step of geometry analysis, we tried to confirm the tetrahedral coordination of the Zn(ii) ion in the imidazole complex. The EXAFS analysis was performed for the Zn(ii)–imidazole spectrum using the model structure presented in Fig. 3A, obtained by optimization calculations as described in the Methods section. The model structure was the same as in *ab initio* calculations. The EXAFS analysis was conducted in the *k*-range of 2.5–12.7 Å^−1^ and the *R*-range of 1.1–4.0 Å. The RBKG parameter was set to 1.0; there were 6 paths used in total. The value of Δ*E*_0_ was found to be 2.85(57) eV; thus, it is correct within the uncertainty level. The first shell is reproduced by 4Zn–N scatters in total: one at 2.098(12) Å and three at 2.013(6) Å (Fig. 4). Further shells are reproduced by single scatters from the closest C atoms and by multiple scattering contributions originating from 1,3-diazole aromatic rings. Interestingly, a strong anharmonic contribution for the first shell Zn–N scatters is present (see Table 2). The ratio of anharmonicity is 1 : 4 for three Zn–N scatterers at 2.098(12) Å and the Zn–N scatterer at 2.013(6) Å, indicating a strong asymmetry of the imidazole ligands' interactions that correlates with the distance between the ligands and the Zn atom. Nevertheless, the EXAFS fit is in line with conclusions drawn from the *ab initio* calculations.

**Table 2:** EXAFS fitting results for the model shown in Fig. 3A. Values in brackets represent total uncertainties. The rows in bold indicates the 1st shell scatters

No.	Scattering path	N^a^	*σ* ^2^ b/Å^2^	*R* + Δ*R*^c^/Å	*C*_3_/Å^3^
**1**	**Zn–N**	**1.3(1)**	**0.0069(4)**	**2.098(12)**	**−0.00464(23)**
**2**	**Zn–N**	**3.3(1)**	**0.0022(1)**	**2.013(6)**	**−0.00117(7)**
3	Zn–C	4.1(1)	0.0024(1)	2.548(4)	
4	Zn–C	3.0(1)	0.0063(3)	2.694(8)	
5	Zn–C–N	5.8(5)	0.0058(10)	3.585(10)	
6	Zn–N–N	2.7(4)	0.0080(16)	4.220(20)	

a Path degeneration (coordination number).

b Debye–Waller factor.

c Fitted distance.

**Fig. 5:**
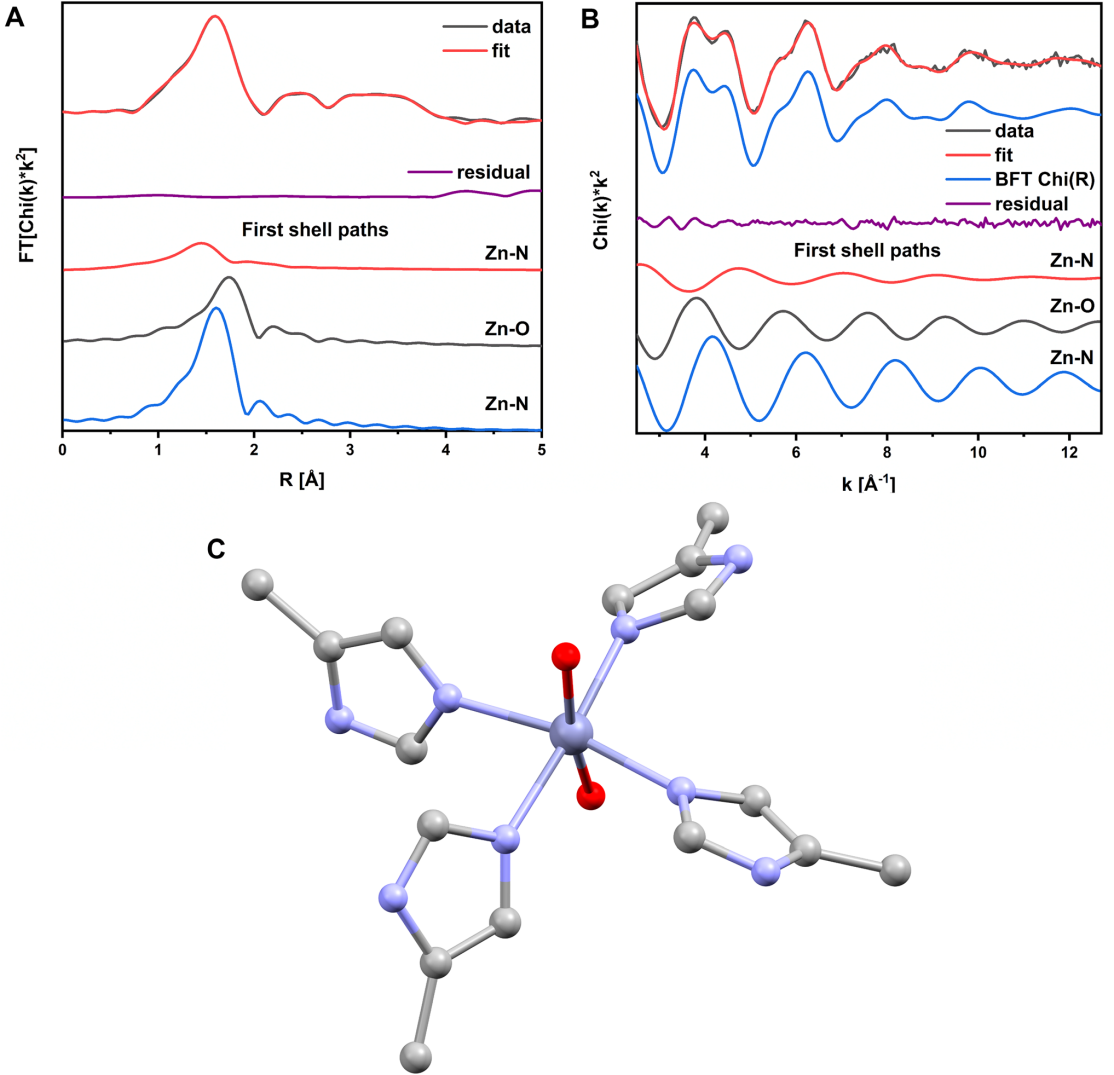
EXAFS fitting results for Zn1 sample with molecular dynamics-derived model: (A) *R* space with Chi(*R*) and fitted function compared to the residual function and first shell paths; (B) *k* space with EXAFS signal (*k*) and fitted function compared to the backward Fourier transform-filtered EXAFS (FFT(Chi(*R*))), residual function and first shell paths; (C) structural model used in fitting.

*Ab initio* calculations in Fig. 2A suggest that Zn(ii) in Zn1 is in the octahedral coordination geometry. A comparison of the experimental data with the results for the Zn(ii)–(MeIm)_6_, and the direct comparison of EXAFS spectra in Fig. 2B suggest that the first shell also contains another type of light atoms, preferably O. This is in line with molecular dynamic simulations performed in our group,^31,58^ where octahedral coordination was proposed, with four N atoms from imidazole rings and two axial water molecules were involved in the complex, as presented in Fig. 5C. The angle between two Zn(ii) bonds varied from 80.8° to 95.2°.^59^ The initial structure was obtained *via* molecular dynamics; however, for the sake of precision, a further optimization was performed in the same way as for the Zn(ii)–Im_4_ model.

### Zn1 EXAFS analysis

The EXAFS analysis of the Zn1 spectrum, assuming the model in Fig. 5C, was performed in the *k*-range of 2.5–12.7 Å^−1^ and the *R*-range of 1.1–4.0 Å. The RBKG parameter was set to 1.0, and 9 paths were used in total for DB calculations. The value of Δ*E*_0_ was found to be 10.15(1.15) eV; thus, it was still correct (10 eV) within the uncertainty level. The total number of the 1st shell ligands was 6, which was consistent with the initial structural model. The first coordination shell signal was reproduced by three scatters: a single degenerated Zn–N path at 1.843(5) Å, a triple degenerated Zn–N path at 1.988(9) Å and a double degenerated Zn–O path at 2.146(1), which reproduced well the 1st coordination shell (Fig. 5). The obtained Zn–N bond length values are characteristic of the distances between N atoms from imidazole rings and Cu(ii)/Zn(ii) ions reported in the literature.^46,58^ The exact values of coordination numbers, positions, and DB factors are presented in Table 3. The final EXAFS fit represents the average coordination numbers for our sample; due to multiple possibilities of Zn(ii) coordination geometries, we cannot exclude that additional chemical environments of Zn(ii) are present. In fact, based on the XANES data in Fig. 1, we expect minor fractions of other Zn(ii) complexes to exist. The planar histidine/imidazole-based structure of the complex in Fig. 5C is the dominating structure, formed and stabilized much more easily in a short peptide chain. This conclusion is in accordance with the MD calculations. The 4N coordination pattern of Zn(ii) ions has been published in many research reports,^17,48^ yet it has mostly tetrahedral geometry, and only in limited cases represent the square planar or pseudo-tetrahedral geometry (regardless of the type of ligands), for example, in human serum albumin.^48,53^ It was shown for the octarepeat peptide that the square-planar conformation of the Zn(ii) complex is possible due to the strong steric interactions of His residues.^46^ As an additional benefit of the planar structure, Zn(ii) can be easily accessed by any other molecule, which literature reports connect with the increased neuroprotective role of PrP^C^–Zn(ii) complexes.^54^ Nevertheless, the EXAFS analysis was also done assuming model structures built in analogy for the corresponding PrP–Cu(ii) complex (Table S1, Fig. S6B, and S7), a common Zn binding motif found in the SOD molecule^60^ (Table S2, Fig. S6A, and S8), and for the planar part of the complex shown in Fig. 5C (Table S3 and Fig. S9). This approach provides a certain level of verification that the Zn1 sample was 6-fold coordinated. The results for the listed models' fits are significantly worse than for the model presented in Fig. 5C due to some non-physical values present in the best possible fits. Details are available in SI. Therefore, we interpret the model in Fig. 5C as the one that reproduces the structure of the Zn(ii) binding site in the PrP^58–91^–Zn(ii) complex. Moreover, the Zn(ii) ion binding motif consisting of four histidines from octarepeats of PrP probed by a Zn(ii) analog- ^113^Cd(ii) NMR spectroscopy was described by Markham *et al.*^61^ and a similar complex of Zn(ii) ions with human PrP was reported in our previous studies.^31,61^

**Table 3:** EXAFS fitting results for the model shown in Fig. 5C. Values in brackets represent total uncertainties. The rows in bold indicates the 1st shell scatters

No.	Scattering path	N^a^	*σ* ^2^ b/Å^2^	*R* + Δ*R*^c^/Å
**1**	**Zn–N**	**0.8(1)**	**0.0055(4)**	**1.843(5)**
**2**	**Zn–N**	**2.7(3)**	**0.0074(7)**	**1.988(9)**
**3**	**Zn–O**	**2.3(1)**	**0.0023(1)**	**2.146(1)**
4	Zn–C–N	3.0(1)	0.0023(1)	2.936(1)
5	Zn–C	0.7(3)	0.0075(24)	3.310(34)
6	Zn–C	5.2(7)	0.0022(9)	3.360(11)
7	Zn–O–O	6.0(4)	0.0025(4)	3.996(5)
8	Zn–N–C	8.0(8)	0.0063(5)	4.215(5)
9	Zn–N	7.4(4)	0.0053(3)	4.427(4)

a Path degeneration (coordination number).

b Debye–Waller factor.

c Fitted distance.

The EXAFS fitting is a powerful tool, yet it does not have to give the final answer. The main limitation of the method is the lack of ligand-type sensitivity for light elements. In this study, we presented the structural model for EXAFS fitting based on the following contexts: (1) the ion to peptide ratio in the PrP^58–91^–Zn(ii) complex; (2) *ab initio* calculations showing that while Zn(ii) coordinated by imidazole is in tetrahedral form, the PrP^58–91^–Zn(ii) complex exhibits octahedral geometry, most probably having axial H_2_O molecules; (3) an optimized, independent result of the molecular dynamic simulation in Fig. 5C which is very similar to our structural model used for calculations of the PrP^58–91^–Zn(ii) complex XANES spectrum. The final EXAFS fit shows that with a high probability, Zn(ii) is coordinated by four imidazole rings in one plane and two axial H_2_O molecules. The requirement of multiple scattering paths in the theoretical description of the given EXAFS signal also indicates the presence of multiple imidazole rings. Moreover, we conclude that this step is characteristic of Zn(ii) binding by PrP^58–91^ peptide with low availability of Zn(ii) ions, as the obtained configuration concerns the 1 : 1 peptide to ion ratio. Finally, the asymmetry in Zn–N distances observed for the 1st coordination sphere can be imposed by the constraints originating from the secondary and tertiary protein structure. To verify this last claim, we have conducted the secondary structure analysis using FTIR spectroscopy.

### Secondary structure analysis

**Fig. 6:**
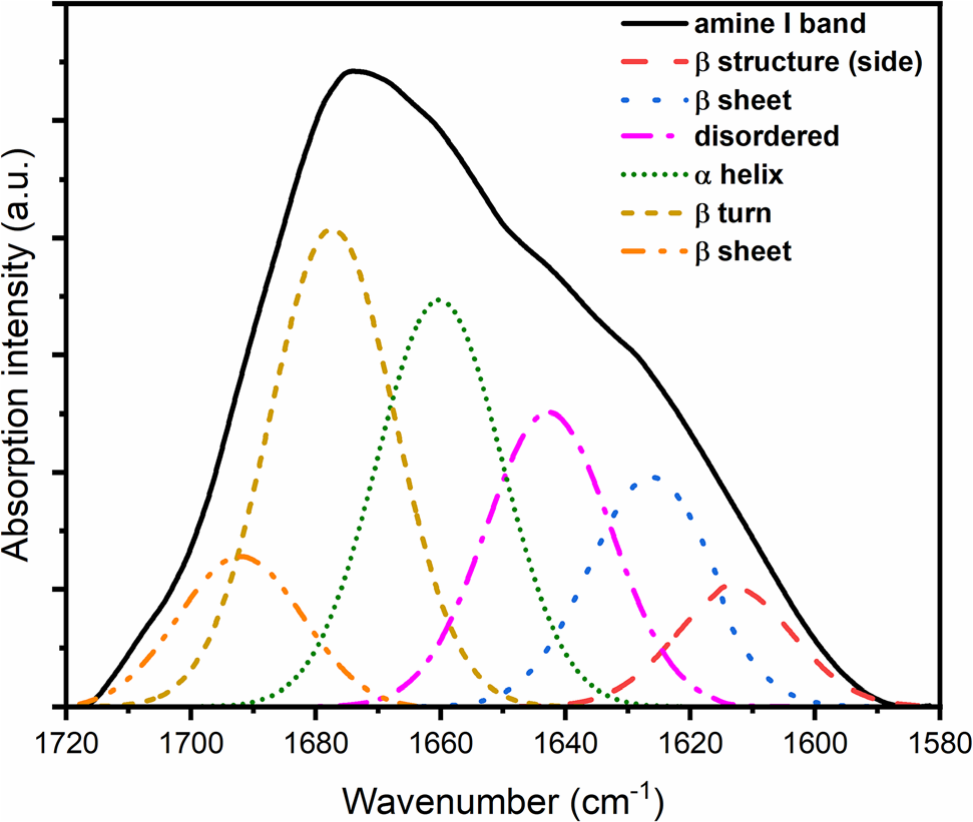
FTIR spectrum of the PrP^58–93^ sample with components obtained from amide I band deconvolution.

**Fig. 7:**
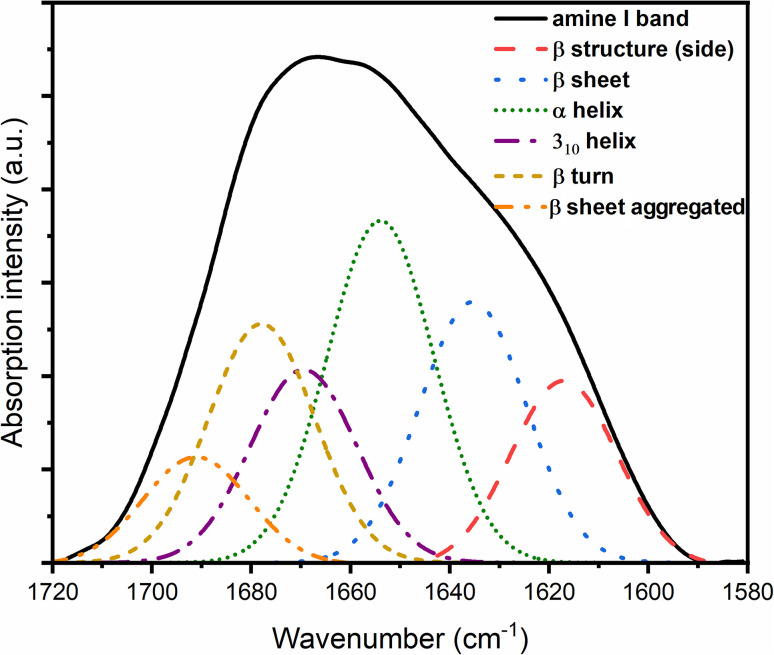
FTIR spectra of PrP^58–93^ and ZnCl_2_ samples at a molar ratio of 1 : 1 with components obtained from amide I band deconvolution.

**Fig. 8:**
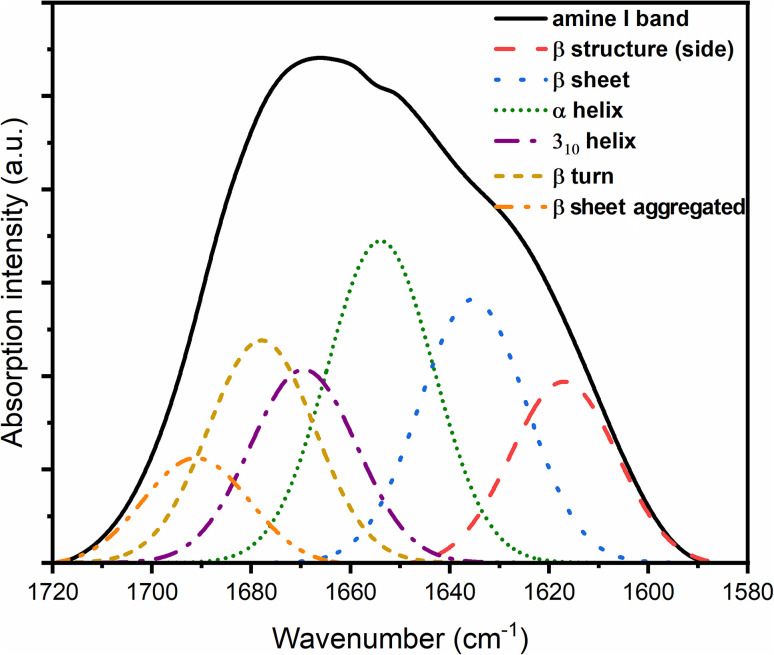
FTIR spectra of PrP^58–93^ and ZnCl_2_ samples at a molar ratio of 1 : 4 with components obtained from amide I band deconvolution.

In addition to the XAS studies, we studied the reference PrP^58–93^ peptide complexes with Zn(ii) ions at 1 : 1 and 1 : 4 stoichiometries by FTIR. The PrP^58–93^ peptide had a sequence longer by 2 amino acids (C-terminal Gly residues), which did not make a significant difference in the complexation of Zn(ii) ions. The FTIR description of the secondary structure of the PrP^58–93^ complexes in the solid-state serves as a reference for the systems tested in this work and, on the other hand, could also be compared to the FTIR results obtained for the solution in the previous study.^32^ The FTIR spectra of the PrP^58–93^ peptide and its complexes with Zn(ii) ions are presented in Fig. 6–8. The deconvolution of the amide I band for the PrP^58–93^ sample showed the presence of a component at 1646 cm^−1^ (Fig. 6, pink line), which corresponds to the band characteristic of disordered protein or peptide fragments.^40,62^

After the addition of Zn(ii) ions, this component (unordered structure) is not present in the amide I band spectra for complexes, while the content of β structure increases (Table 4), which shows the influence of Zn(ii) ions and the increase in the ordering of the structure of the prion protein peptide. In addition, after deconvolution performed for the PrP^58–93^ : Zn(ii) 1 : 1 (Fig. 7) and PrP^58–93^:Zn(ii) 1 : 4 samples (Fig. 8), the band at 1663 cm^−1^, which could be ascribed to a 3_10_ helix structure, showed a reduced intensity.^40,62^ Previous studies have shown that the dominant secondary structure for the octarepeat fragment of the prion peptide is a random coil and polyproline II helix (PPII), as revealed by circular dichroism spectroscopy.^31,32,63–65^ In this study, deconvolution of the ATR-FTIR spectra of pure PrP^51–91^ peptide deposits showed that even the sample without the addition of Zn(ii) ions shows some level of ordering. The peaks at 1627 cm^−1^, 1662 cm^−1^, and 1678 cm^−1^, conventionally assigned to β-sheet, 3_10_-helix, and β-turn, respectively, can be interpreted together as PPII helix,^66^ which is consistent with our previous results.^31,32^ After the addition of Zn(ii) ions, the intensity of bands at 1662 cm^−1^ and 1678 cm^−1^ decreased, and the intensity of bands at 1618 cm^−1^ and 1627 cm^−1^ increased. This result can be interpreted as a reduction of PPII helix structure and formation of β-sheet structure, what is supported by our previous results from circular dichroism spectroscopy.^31^ On the other hand, solid-state FTIR studies of a number of proteins and peptides show a higher content of organized structures in comparison to liquid sample spectra.^67,68^ For proteins that are rich in α-helix structures or have an intrinsically disordered fragment, the percentage of β structure calculated from the deconvolution of ATR spectra is higher for solid-state samples. This difference is strongly connected with the dehydration of protein during sample drying. When the sample is rehydrated, the secondary structure is restored to that characteristic of liquid samples.^68^ This is why random coil is not the main secondary structure of solid-state PrP samples. A similar dependence was also obtained from the circular dichroism studies of proteins associated in thin films. The random-coil conformation of heterooligomeric peptides and poly(l-lysine) in solutions changes into β-structure and β-turns when deposited on glass or mica surfaces.^67,69^ Even if the β-structure of the PrP sample can be identified from the ATR spectra, it increases significantly in the presence of Zn(ii). This change suggests that after the addition of Zn(ii), the aggregation process can occur, in agreement with our earlier results.^31,32^ The transition from the native state to a β-rich structure is typical for neurodegenerative processes, such as Alzheimer's disease or Parkinson's disease.^70,71^

**Table 4:** The composition of the secondary structure elements determined from the amide I band for studied prion peptide samples

Sample	α-helix	β-sheet	3_10_ helix	β-turns	Disor-dered
Prp^58–93^	24%	29%	—	28%	19%
Prp^58–93^ : Zn^2+^ 1 : 1	22%	40%	13%	25%	—
Prp^58–93^ : Zn^2+^ 1 : 4	21%	45%	16%	18%	—

One should point out that the whole protein contains also C-terminal domain, rich in α-helices and forming a globular shape. It is insoluble in aqueous solutions and the C-terminal domain acts as an anchor, placing PrP in the lipid membrane, with N-terminal part exposed outside.^72^ This, and possible post-translational modifications could affect the aggregation process and structure of the PrP–Zn(ii) complex. Therefore, the experiment should be extended in future for the full PrP protein, preferably anchored in the lipid membrane to account for tertiary structure and anchoring effects and put the results in more biological context.

The literature in not coherent, regarding the actual affinity constants of PrP^c^ as a whole and its octarepeat motif for Cu(ii) and Zn(ii) ions. However, there is no doubt that the Zn(ii) affinity to this motif is weaker than that of Cu(ii) by several orders of magnitude under hypothetical equilibrium conditions.^17,73^ Such direct competition experiments were done and revealed the significant plasticity of the octarepeat motif. At some Cu(ii)/Zn(ii) ratios the simultaneous Cu(ii) and Zn(ii) binding was observed.^73^ However, the methodology applied to Zn(ii) in those studies was indirect, with no structural information; the Zn(ii) binding was inferred from the alteration of Cu(ii)-related signals. It seems that mixed Zn(ii)/Cu(ii) complexes at octarepeats might be formed during neurotransmission.

Finally, the entry of Zn(ii) ions to the synaptic cleft precedes that of Cu(ii) ions.^74^ This requires investigating the kinetics of Zn(ii) binding to octarepeats and stipulates experimental designs in which the preformed Zn(ii)–octarepeats complex is reacted with Cu(ii) ions. Such research is in progress and will be supplemented by XAS studies of combined Zn(ii)/Cu(ii) complexes as a follow-up to the current work.

## Conclusions

The function of intrinsically disordered proteins and protein regions is a key topic in protein research.^75^ The intrinsically disordered PrP^58–93^ peptide has a unique affinity for Cu(ii) and Zn(ii) ions that is strictly connected with the function of the final complexes. Although in various Zn(ii) to PrP stoichiometries, the complex forms different macroscopic aggregates and fibrillar structures, the basal complex geometry is the same in all cases. In this study, we have shown that the local structure of PrP^58–93^–Zn(ii) fibrils can be determined by means of EXAFS experiments, theoretical calculations, and fitting. Based on initial *ab initio* FDMX calculations for PrP^58–93^–Zn(ii) and Zn(ii)–(MeIm)_4_ complex, two different coordination geometries were proposed for the Zn(ii) chemical environment. For PrP^58–93^–Zn(ii) in a 1 : 1 Zn(ii) to PrP ratio, the best fit results indicated the Zn(ii) coordination by four imidazole nitrogens in a planar arrangement, complemented by two axial H_2_O molecules to provide a distorted octahedral geometry. The identified local Zn(ii) geometric asymmetry in Zn–N bond lengths requires a highly organized second-order structure of the peptide, which can involve reduction of PPII helix and the formation of β-structure. Such a hydrophobic structure efficiently promotes oligomerization in aqueous solution, which could initiate the early stage of neurodegenerative disease. The results show that, like Cu(ii), Zn(ii) ions can promote β-sheet formation in the octarepeat peptide, characteristic for the amyloid state. *In vivo* α- and β-cleavage of PrP^C^ yields fragments of N-terminal domain,^76^ which could adopt such structures in living organisms. Pathological PrP variant Y145Stop, associated with genetic form of transmissible spongiform encephalopathy^77^ incubated with Cu(ii) adopts fibrils with an altered core structure.^78^ However, in that case alteration of the core structure was attributed to Cu(ii) binding by C-terminal histidine residues (H96, H111, H140). Whether fibril formation by the octarepeat region contributes to prion pathology and propagation remains an open question and must be further tested *in vitro* and *in vivo* models.

## Author contributions

Conceptualization, M. K., I. Z.; methodology, M. K., M. N. and W. M. K.; validation, A. P., A. S., M. W., I. Z., J. W., J. Ż., M. G., M. K., M. N., W. B., S. W. and W. M. K.; formal analysis, J. W., M. N.; investigation, J. W., M. G., M. K., M. N., M. D. W., W. M. K; resources, A. S., I. Z., M. W., M. K., M. N., and M. G.; data curation, J. W., M. K. and M. N.; writing—original draft preparation, A. P., J. W., M. K., M. N.; writing—review and editing, A. P., A. S., M. G., J. W., I. Z. J. Ż. M. K., M. N., W. B., S. W. and W. M. K.; visualization, J. W. and M. N.; supervision, M. K., M. N. and W. M. K.; project administration, M. K. and W. M. K.; funding acquisition, M. K. All authors have read and agreed to the published version of the manuscript.

## Conflicts of interest

There are no conflicts to declare.

## Supplementary Material

RA-015-D5RA04584C-s001

## Data Availability

The data supporting this article have been included as part of the supplementary information (SI). Supplementary information is available. See DOI: https://doi.org/10.1039/d5ra04584c.
