# Supplementary material for: The structure of complexes between zinc(ii) cations and histidine-rich repeats from the unstructured N-terminal domain of human prion protein
Source: RSC Adv. 2025 Oct 15;15(46):38422–34. doi: 10.1039/d5ra04584c (PMC12522110; doi:10.1039/d5ra04584c)
Supplement: RA-015-D5RA04584C-s001 [file RA-015-D5RA04584C-s001.pdf]

Supplementary Information (SI) for RSC Advances.
This journal is © The Royal Society of Chemistry 2025

Supplementary Information

The electronic structure of complexes between zinc(II) cations and histidine-rich repeats from the unstructured N-terminal domain of human prion protein

Michał Nowakowski[a*], Joanna Wolak[b], Maciej Gielnik[c], Adam Piotrowski[b], Igor Zhukov[d], Justyna Żygowska[e], Aneta Szymańska[e], Marta D. Wiśniewska[d], Wojciech Bal[d], Sebastian K. T. S. Wärmländer[f,g], Maciej Kozak[b,f*], Wojciech M. Kwiatek[g]

[a] *Department of Chemistry, and Center for Sustainable Systems Design (CSSD), Faculty of Science, Paderborn University, Warburger Straße 100, 33098 Paderborn, Germany.*
[b] *Faculty of Physics, Adam Mickiewicz University, Uniwersytetu Poznańskiego 2, Poznań, 61-614, Poland.*
[c] *Department of Molecular Biology and Genetics, Aarhus University, Nordre Ringgade 1, 8000 Aarhus, Dennmark*
[d] *Institute of Biochemistry and Biophysics Polish Academy of Sciences, Pawińskiego 5a, 02-106 Warsaw, Poland*
[e] *Department of Chemistry, University of Gdańsk, Jana Bażyńskiego 8, 80-309 Gdańsk, Poland*
[f] *Chemistry Section, Arrhenius Laboratories, Stockholm University, 106 91 Stockholm, Sweden*
[g] *CellPept Sweden AB, Kvarngatan 10B, 118 47 Stockholm, Sweden*
[h] *Smaug beamline, SOLAIS National Synchrotron Radiation Centre, Czerwone Maki 98, 30-392 Cracow, Poland*
[i] *Institute of Nuclear Physics Polish Academy of Science, Radzikowskiego 152, 31-342 Cracow, Poland*
* Corresponding authors.

Supplementary Information list of contents:

1) Radiation damage study
2) Qualitative XANES analysis
    a) XAS data
    b) Absorption edge position
3) LCF analysis :
    a) LCF fitting
    b) The HSA-Zn(II) reference
4) EXAFS analysis

1) Radiation damage studies

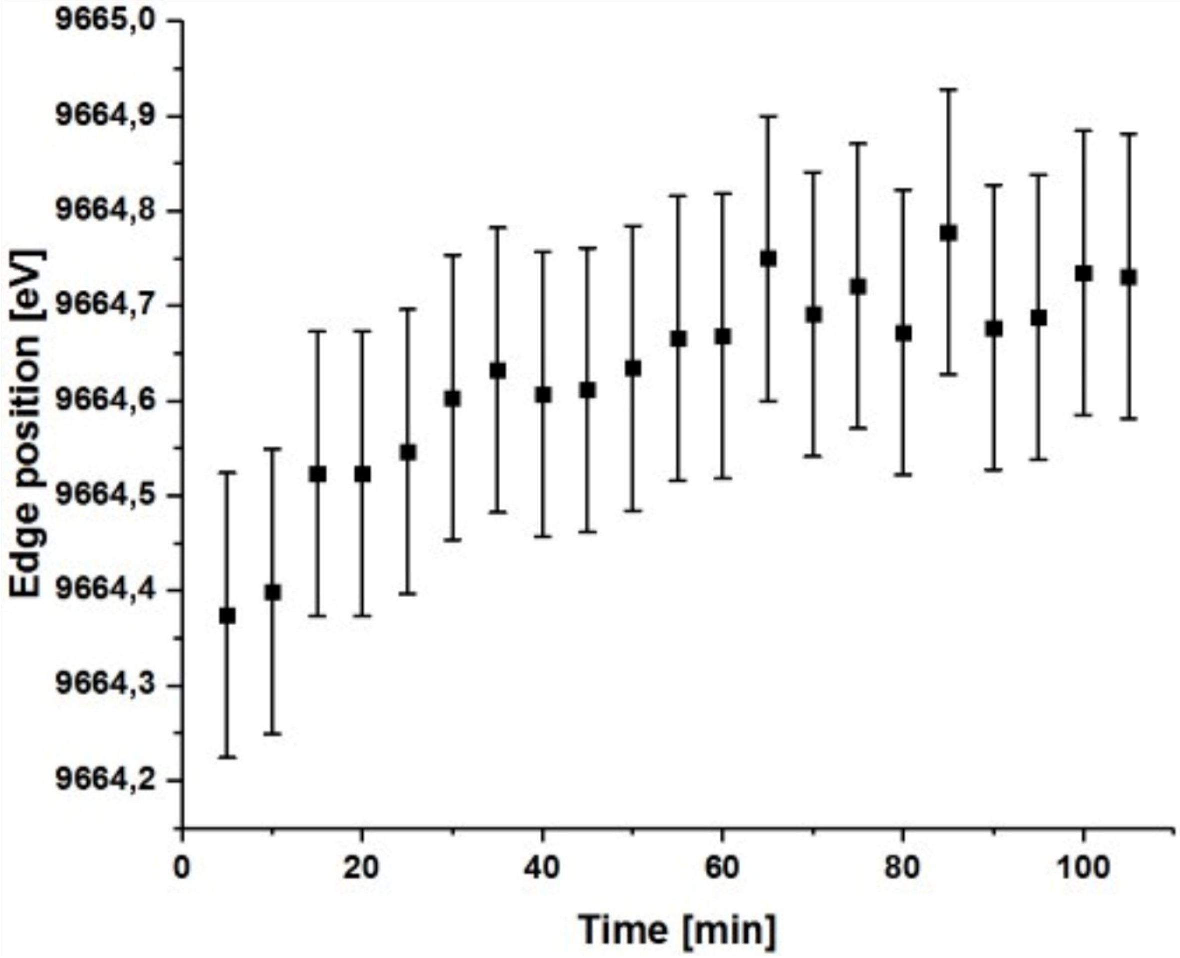

Fig. S1 The radiation damage study. **Zn1** sample was irradiated in one spot for extended time (approximately 2 h) and after each 5 minutes, a short XANES scan was performed. E_0 was read at first value equal to 0 in 2nd derivative. The uncertainty value equals 0.15 eV as indicated in the text. The above data indicate weak growing trend, which is at the end saturated, however in the range of uncertainty no significant radiation damage is visible.

2) Qualitative XANES analysis

a) XAS data

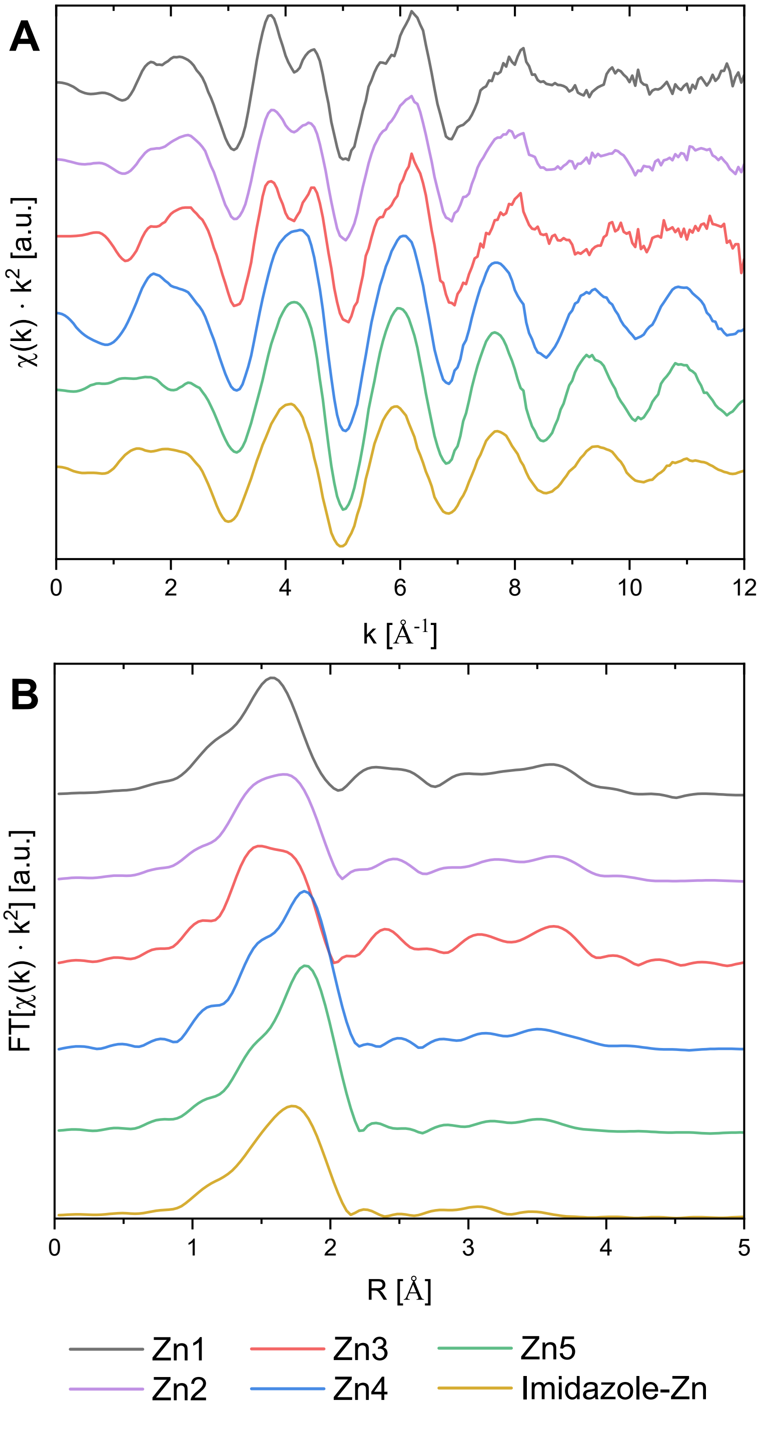

Fig. S2. The EXAFS data of PrP$^{58\text{-}91}$ -Zn(II) 1-5: A – Chi(k)*k^2 signal; B – Fourier Transform of Chi(k)*k^2 signal (Chi(R))

b) **Absorption edge position**

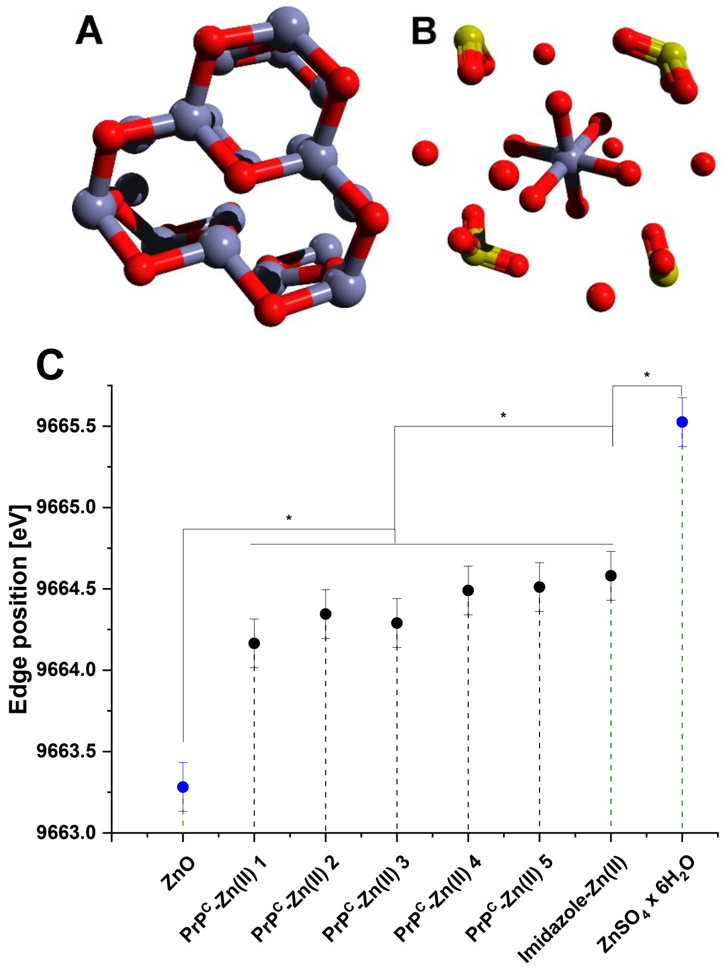

Fig. S3. A - Tight, ordered structure of wurtzite (ZnO); **B** – 3D structure of $ZnSO_4$ x $7H_2O$; **C** - Edge position in function of all measured compounds. Neighbouring groups of points, which are statistically significantly different from each other, were marked by * and different colour. Statistical difference was evaluated using t-Student test with statistical significance $p < 0.05$.

3) LCF analysis

a) LC fitting

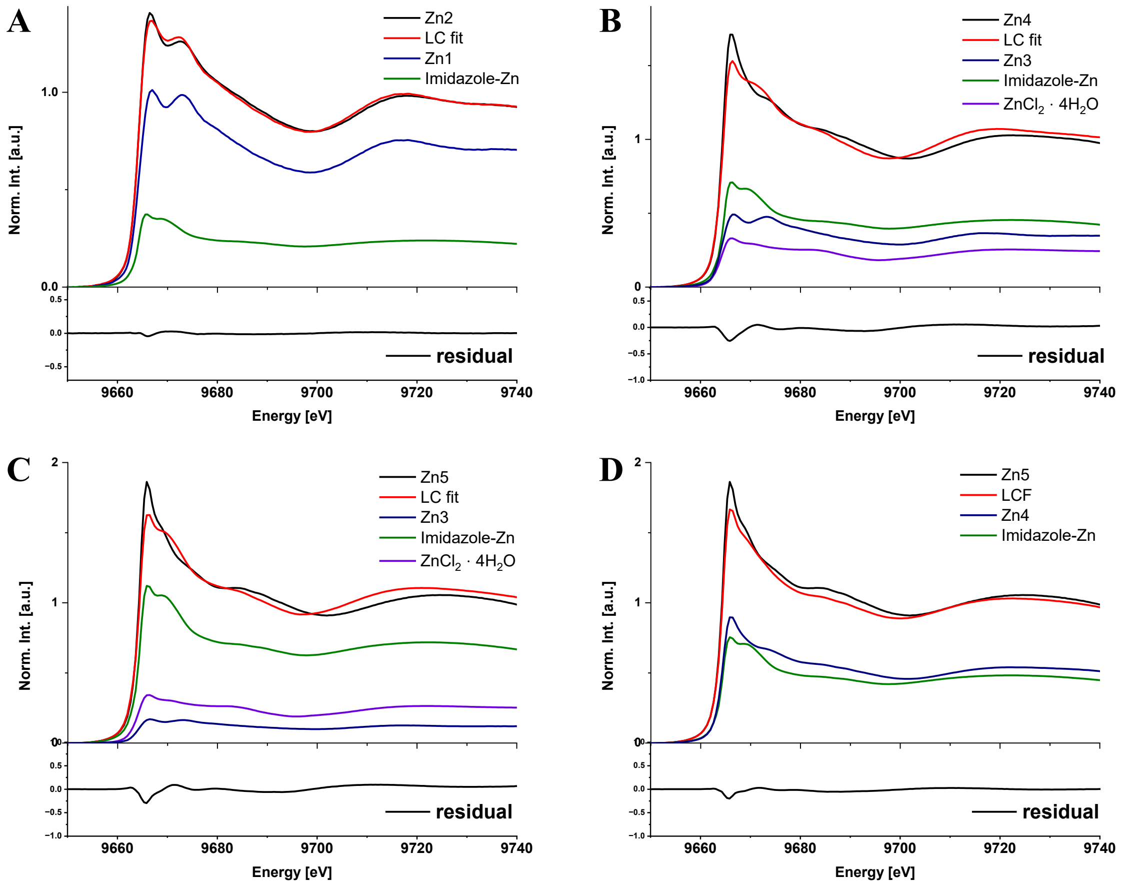

Fig. S4. LCF results for: **A** – PrP$^{58\text{-}91}$-Zn(II) 2; **B** – PrP$^{58\text{-}91}$-Zn(II) 4; **C** – PrP$^{58\text{-}91}$-Zn(II) 5 fitted with PrP$^{58\text{-}91}$-Zn(II) 3 spectrum; **D** – PrP$^{58\text{-}91}$-Zn(II) 5 fitted with PrP$^{58\text{-}91}$-Zn(II) 4 spectrum. Organic-Zn(II) denotes the HAS-Zn(II) sample. Since protein was measured in solution, $ZnCl_2$ was used in the form of $[Zn(OH)_4]^{2-}$.

b) The HSA-Zn(II) reference

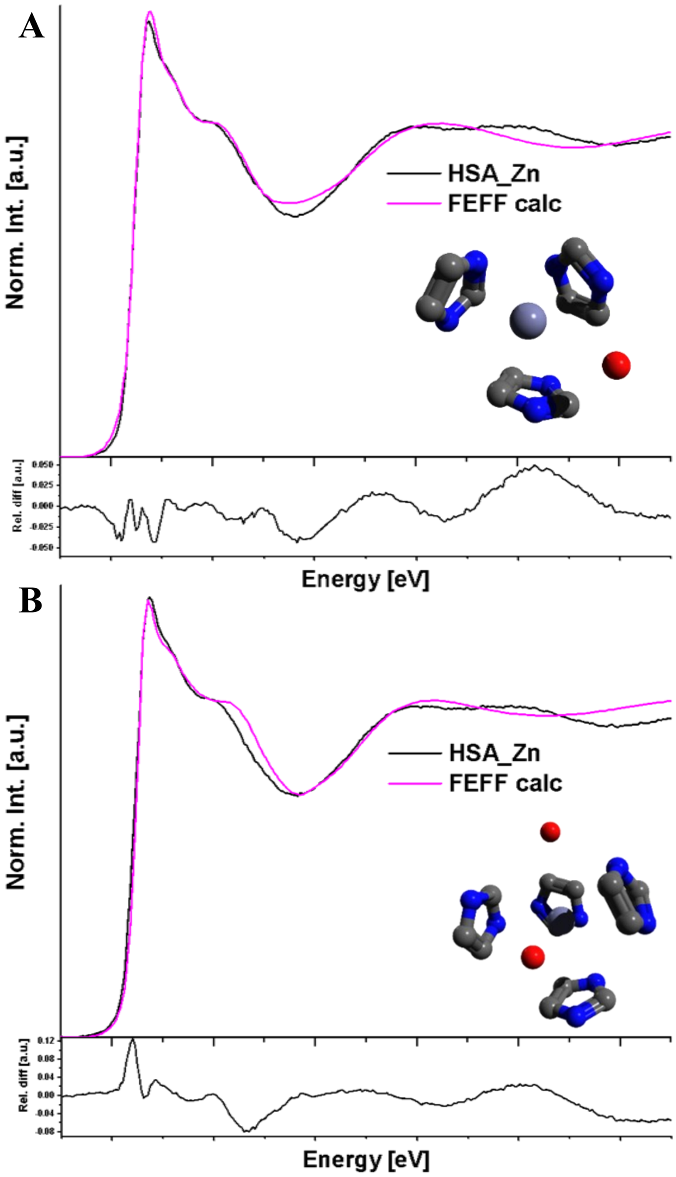

Fig. S5. Characterization of the HSA-Zn(II) sample: **a)** 3N + O geometry and **b)** 4N+O geometry with additional water molecule .

The initial structures were built in the Avogadro software and further optimized with the use of the Universal Force Field and then refined with DFT approach, as described in the Methods section. The two proposed structures are presented as inserts in parts A and B. X-Ray calculations were performed in FEFF 9.6 software with the use of the Full Multiple Scattering with Self Consistency Field for the muffin-tin approximation. Additional Heidin-Lundqvist exchange-correlation potential with ground state background function was applied. The core hole screening was modelled with Final State Rule approach. No Fermi level shift was applied. Bottom of Figs S5 A-B present a

difference between theoretical and experimental spectra. On the basis of the differential plot, one can assume that the edge position was reproduced better in B picture than in A figure.

5) EXAFS analysis

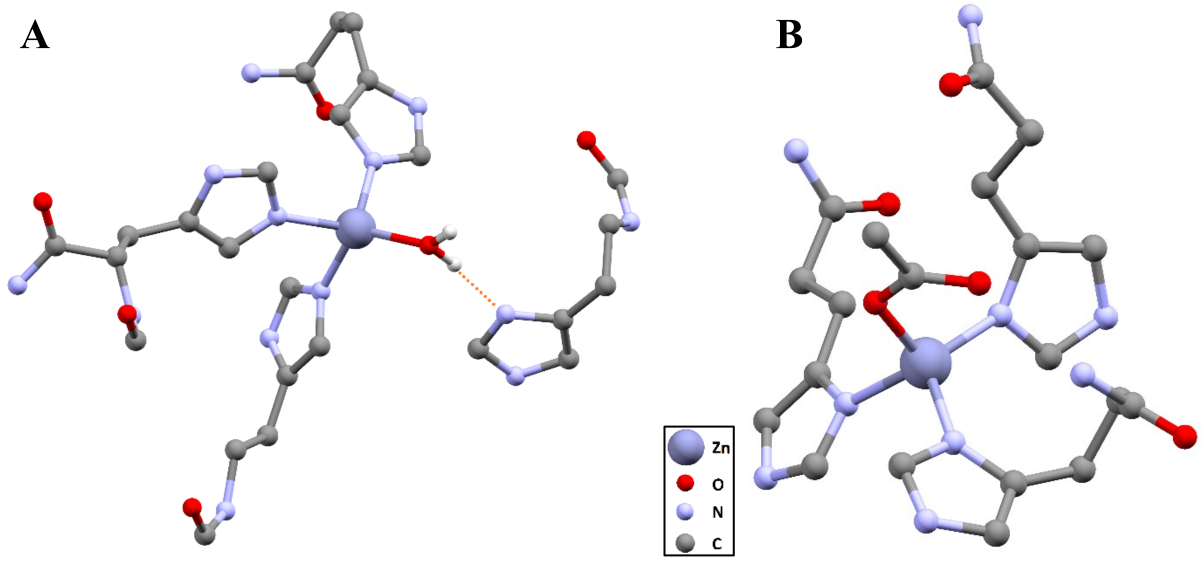

Fig. S6. Two structures of PrP$^{58\text{-}91}$-Zn(II) complex used in this study: **A** - DFT optimized model of PrP$^{58\text{-}91}$-Zn(II) complex with constitutional H_2O molecule, in analogy to *hu*PrP^{C}-Cu(II) model; **B** - Zn(II) binding site in Superoxide Dismutase (1ozu.pdb). Hydrogen atoms were omitted for clarity.

The analysis assuming the model in Fig. S6 A was performed in the k-range of 2.0 – 13.0 Å^{-1} and 1.2 – 4.5 Å R-range. The value of E_0 for PrP$^{58\text{-}91}$-Zn(II) 1 sample was set to 9664.17 eV and RBKG value was equal to 1. For MS calculations, FEFF 7 was used. EXAFS analysis was performed in R space and in total 12 paths, both single and multiple scattering were used. For Debye- Waller (DB) factors calculations of single scattering (SS) paths, the best EXAFS fitting result to the Einstein model was used (1). The uncertainties of DB factors were obtained by applying the exact differential method to equation (1) which defines the DB factor in the Einstein model:

$$\sigma_E\,(T)=\hbar/(2\mu\omega_E\,)\,\coth(\,(\hbar\omega_E)/(2kT)) \qquad (1)$$

where: $\hbar$ - Dirac constant; μ – reduced mass of atoms engaged in scattering process; ω_E – Einstein frequency; k – Boltzmann constant; T – temperature. In general, the Einstein model is valid when applied to systems with one or two types of atoms. However, in EXAFS, light atoms are not distinguishable. This also translates into the DB factor defined by eq. (1), since its value depends on reduced mass, which, on the other hand, is dominated by the Zn contribution. For example, the reduced mass corresponding to $SS_{Zn\text{–}N}$ is equal to the 9.5 u, while that of $SS_{Zn\text{–}O}$ it

is 10.4 u. Therefore, all light atoms in the structure presented in Fig. S6 A can be reasonably approximated by one type of atoms within 10 % uncertainty, which, in consequence, justifies the application of the Einstein model. The amplitude reduction factor, due to multi-electron excitations (SO_2), was set to 1.0 and temperature in the Einstein model was set to 10 K. The ΔE_0 value, obtained in the fit, was equal to 4.811(658) eV and the frequency in the Einstein model was fitted with the final value of 1087 ∓ 293 kHz. No correlations over 0.95 between any of the parameters were observed. Statistical evaluations of R and reduced $\chi 2$ parameters were 0.011 and 61.86 respectively. Detail values for all paths are shown in Table S1. According to the fitting results, the 1st coordination shell is composed of 5 atoms, corresponding to 3 main single scatters (Fig. S7 A-B). It is especially well visible in Fig S7 B, where the first peak between 1 -2 Å is reproduced. As EXAFS amplitude is very sensitive to coordination number values, for refinement, all of the N values were re-fitted simultaneously to avoid artifacts generation. The differences between the obtained values and positions in the figure are due to phase shifts that are not included in the plotted figures.

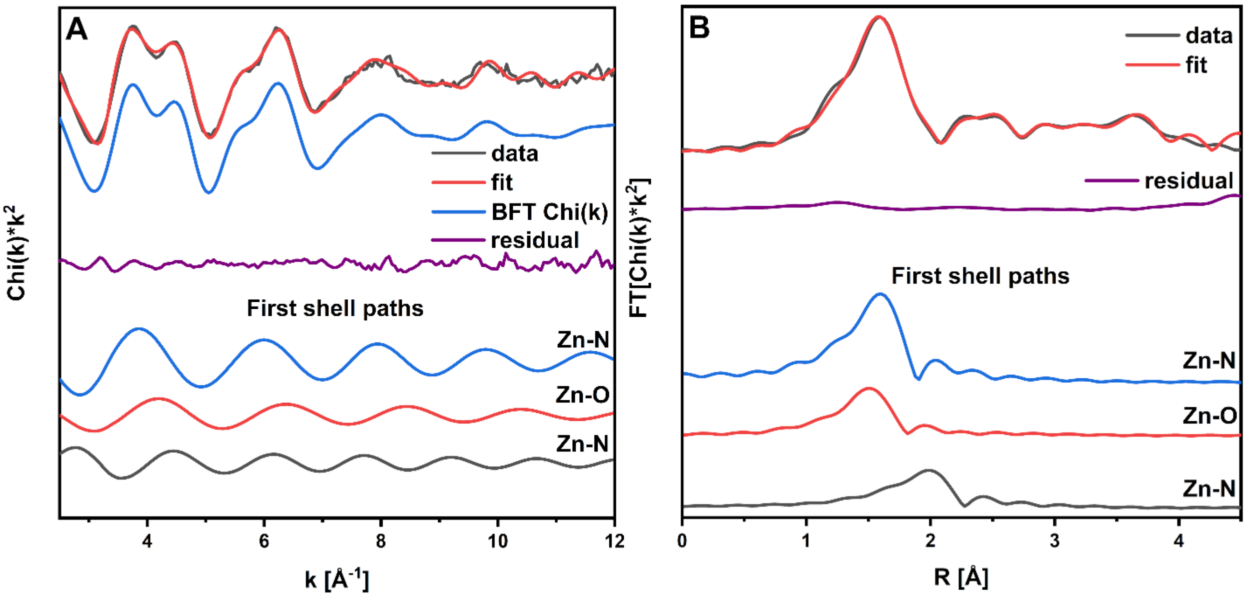

Fig. S7 EXAFS fitting results for PrP58-91-Zn(II) 1 sample with initial model taken from DFT optimized model of PrP58-91-Zn(II) complex with constitutional H_2O molecule : **A** – k space with EXAFS signal (k) and fitted function compared to the backward Fourier filtered EXAFS (FFT(Chi(R)), residual function and first shell paths; **B** – R space with FT(Chi(R)) and fitted function compared to the residual function and first shell paths.

The closest atom is represented by SS_{Zn-O} at 1.919(14) Å and the furthest 2 N atoms are represented by SS_{Zn-N} at 2.475(12) Å. While they are denoted and included into Path Expansion as contributions from O and N atom, as mentioned above, EXAFS does not distinguish between light elements such as C, N, or O. Therefore, the notation which distinguishes these atoms is only arbitrary in accordance to the initial fitting model. Based on the values of coordination numbers for this refined $PrP^{58\text{-}91}$-Zn(II) structure, one can assume that SS_{Zn-O} at 1.919(14) Å represents the oxygen atom from the constitutional H_2O molecule.

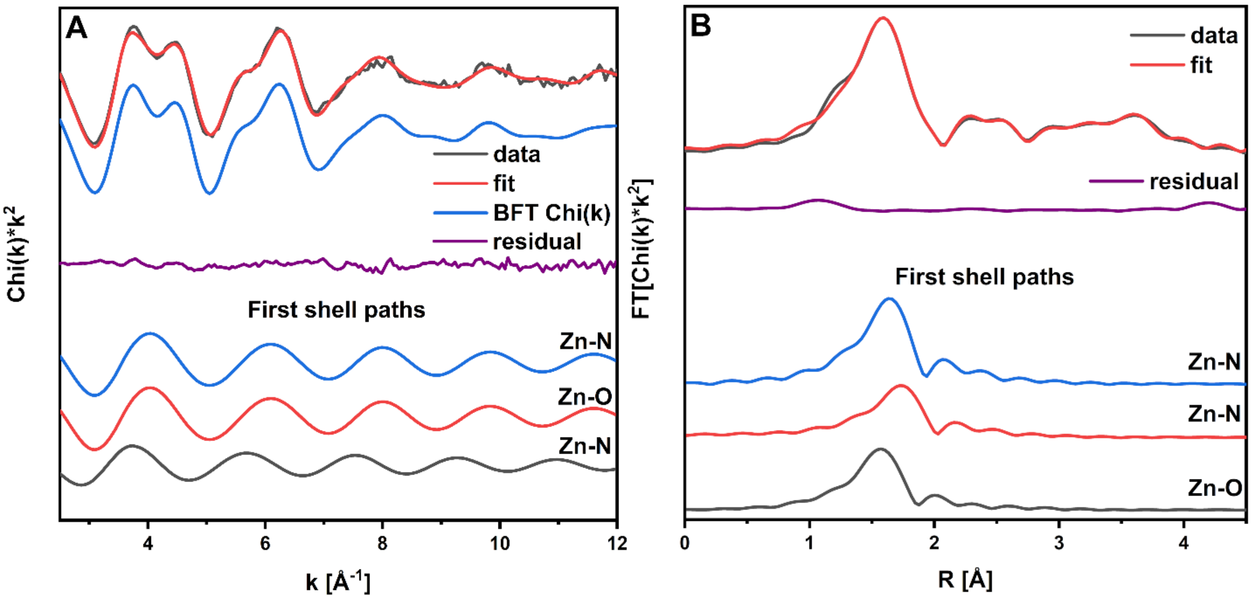

Fig. S8 EXAFS fitting results for $PrP^{58\text{-}91}$-Zn(II) 1 sample with initial model taken from 1ozu.pdb structure: **A** – k space with EXAFS signal (k) and fitted function compared to the backward Fourier filtered EXAFS (FFT(Chi(R)), residual function and first shell paths; **B** – R space with FT(Chi(R)) and fitted function compared to the residual function and first shell paths.

The EXAFS analysis assuming the model in Fig. S6 B was performed in the k-range of 2.0 – 13.0 $Å^{-1}$ and 1.2 – 4.5 Å of the R-range. The rest of parameters, number of paths and DB calculations were the same as in the analysis of model A. The ΔE_0 was found to be 11.01(2.02) eV, thus still in the commonly accepted range of ± 10 eV when uncertainty is included. The frequency in the Einstein model was fitted with the final value of 1664 ∓ 340 kHz. No correlations over 0.95 between any of the fitted parameters occurred. Statistical evaluations of R and reduced $\chi 2$ parameters were o 0.007 and 46.02 respectively, which are better values than those corresponding to model A. It can be seen in Fig. S8, that the first 3 paths well reproduced the main peak in $FT(Chi(k)*k^2)$ signal. The total number of the 1st shell ligands is 4 in accordance to the initial

structural model. The closest atom is O characterized by SS_{Zn-O} at a distance of 1.956(8) Å. Next three atoms are represented by two SS_{Zn-N} scatterers at distances and coordination numbers of 2.043 Å, N ≈ 1 and 2.146 Å, N ≈ 2, respectively.

Table S1. EXAFS fitting results for the model shown in Fig. S6 A. Values in the brackets represent total uncertainties. The grey area indicates the 1st shell scatters.

No.	Path	N	σ^2	R+ΔR / Å
1	Zn-N	1.7(2)	0.0019(7)	2.475(12)
2	Zn-O	0.9(1)	0.0017(6)	1.919(14)
3	Zn-N	2.4(1)	0.0019(6)	2.036(6)
4	Zn-C	5.4(2.1)	0.0022(7)	3.466(28)
5	Zn-C-N	4.4(3.8)	0.0056(10)	3.187(47)
6	Zn-N	7.4(1.1)	0.0039(12)	4.253(17)
7	Zn-C-N	5.6(1.1)	0.0057(11)	2.545(30)
8	Zn-C	4.8(1.9)	0.0023(8)	3.617(35)
9	Zn-N-C	15.0(5.6)	0.0096(16)	3.631(29)
10	Zn-C	11.7(1.2)	0.0033(11)	4.833(21)
11	Zn-C	2.7(6)	0.0021(7)	2.980(16)
12	Zn-C	1.6(1.1)	0.0037(12)	3.906(47)

Table S2. EXAFS fitting results for the model shown in Fig. S6 B. Values in the brackets represent total uncertainties. The grey area indicates the 1st shell scatters.

No.	Path	N	σ^2	R+ΔR / Å
1	Zn-O	1.0(1)	0.0011(2)	1.956(8)
2	Zn-N	1.3(1)	0.0013(2)	2.146(7)
3	Zn-N	1.9(2)	0.0013(2)	2.043(6)
4	Zn-C	1.9(3)	0.0014(2)	3.023(14)
5	Zn-C	1.4(2)	0.0015(2)	2.544(11)
6	Zn-N-C	30.7(10.3)	0.0037(3)	4.214(50)
7	Zn-C	13.0(1.7)	0.0016(3)	3.990(14)
8	Zn-C-N	4.1(3.0)	0.0043(4)	3.562(142)
9	Zn-C	2.6(1.0)	0.0018(3)	3.747(21)
10	Zn-O	3.9(7)	0.0023(4)	3.798(25)
11	Zn-C	26.4(3.2)	0.0021(3)	4.183(10)
12	Zn-N-N	30.7(5.5)	0.0046(4)	4.264(17)

The EXAFS analysis assuming the model in Fig. S9 was performed in the k-range of 2.5–13.0 Å-1 and the R-range of 1.25–4.0 Å. The RBKG parameter was set to 1.2, there were 11 paths used in total, and for DB calculations, the Einstein model was not used this time. The value of ΔE_0 was found to be 10.01(49) eV; thus, it was still correct (10 eV) within the uncertainty level. The evaluations of R and reduced $\chi 2$ parameters gave values of 0.002 and 19.68, respectively, which were the best results in terms of the statistical quality of the fit. The first two Zn-N paths with coordination numbers of 2 each and at distances of 2.000(4) Å and 2.126(6) Å reproduced well the 1st coordination shell (Fig. S9 B) and dominated the EXAFS signal (Fig. S9 A). The total number of the 1st shell ligands was 4, which was consistent with the initial structural model. The obtained Zn-N bond length values are characteristic of the distances between N atoms from imidazole rings and Cu(II)/Zn(II) ions reported in the literature (2,3). The exact values of coordination numbers, positions and DB factors are presented in Table S3. This fitting result contains the smallest uncertainties of the fitted values and the best overall fit evaluation in terms of the parameters R and reduced $\chi 2$.

Table S3. EXAFS fitting results for the model shown in Fig. S9 C. Values in brackets represent total uncertainties. The grey area indicates the 1st shell scatters.

No.	Scattering path	N	σ^2	R+ΔR/Å
1	Zn-N	2.2(1)	0.0011(3)	2.000(4)
2	Zn-N	1.8(1)	0.0011(4)	2.126(6)
3	Zn-C	4.7(7)	0.0120(9)	2.595(15)
4	Zn-C	13.3(3.3)	0.0120(9)	3.070(12)
5	Zn-C	16.0(2.8)	0.0123(10)	3.309(15)
6	Zn-C-N	12.8(1.6)	0.0126(11)	2.709(27)
7	Zn-N	18.1(8)	0.0021(7)	4.299(11)
8	Zn-N-N	19.6(9)	0.0016(5)	4.394(13)
9	Zn-C-N	21.4(2.8)	0.0126(11)	3.380(17)
10	Zn-C-N	29.0(8.9)	0.0126(11)	3.041(34)
11	Zn-C	2.9(1.4)	0.0169(13)	3.966(50)

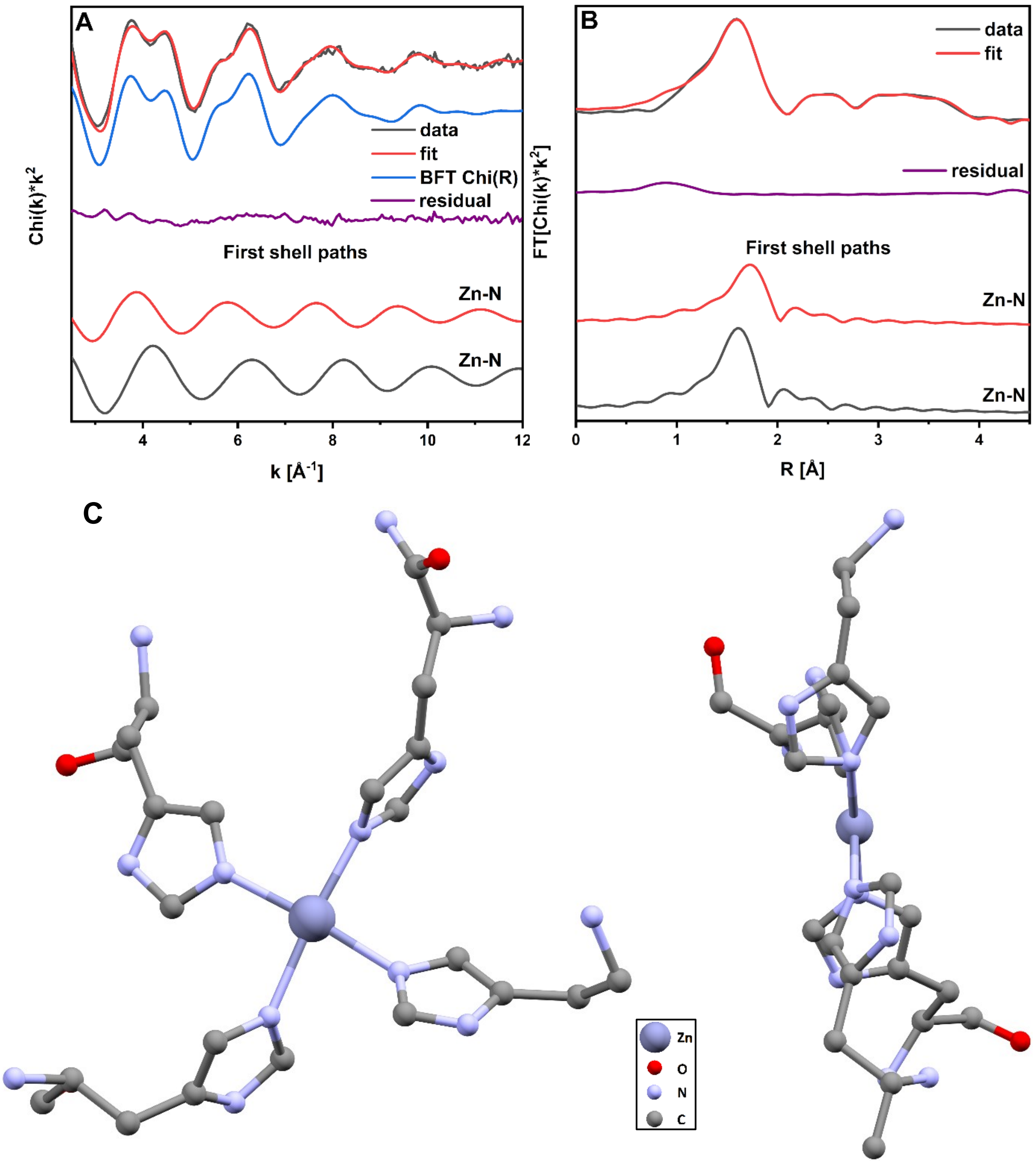

Fig. S9. EXAFS fitting results for PrP58-91-Zn(II) 1 sample with molecular mechanics-derived model: **(a)** k space with EXAFS signal (k) and fitted function compared to the backward Fourier transform-filtered EXAFS (FFT(Chi(R)), residual function and first shell paths; **(b)** R space with Chi(R) and fitted function compared to the residual function and first shell paths; **(c)** structural model used in fitting (front and side view).

References

1. Dalba G, Fornasini P. EXAFS Debye - Waller factor and thermal vibrations of crystals. Journal of Synchrotron Radiation. 1997;4(4):243–55.

2. Pushie MJ, Nienaber KH, McDonald A, Millhauser GL, George GN. Combined EXAFS and DFT structure calculations provide structural insights into the 1:1 multi-histidine complexes of Cu(II) , Cu(I) , and Zn(II) with the tandem octarepeats of the mammalian prion protein. Chemistry (Weinheim an der Bergstrasse, Germany). 2014;20(31):9770–83.

3. McDonald A, Pushie MJ, Millhauser GL, George GN. New insights into metal interactions with the prion protein: EXAFS analysis and structure calculations of copper binding to a single octarepeat from the prion protein. Journal of Physical Chemistry B. 2013;117(44):13822–41.
